# Mutations in XRCC1 cause cerebellar ataxia and peripheral neuropathy

**DOI:** 10.1136/jnnp-2017-317581

**Published:** 2018-02-22

**Authors:** Emer O’Connor, Jana Vandrovcova, Enrico Bugiardini, Viorica Chelban, Andreea Manole, Indran Davagnanam, Sarah Wiethoff, Alan Pittman, David S Lynch, Stephanie Efthymiou, Silvia Marino, Adnan Y Manzur, Mark Roberts, Michael G Hanna, Henry Houlden, Emma Matthews, Nicholas W Wood

**Affiliations:** 1 Department of Molecular Neuroscience, Institute of Neurology, University College London, London, UK; 2 Department of Brain Repair and Rehabilitation, Institute of Neurology, University College London, London, UK; 3 Department of Neuropathology, Barts and The London School of Medicine and Dentistry, Queen Mary University of London, London, UK; 4 Department of Neurology, Great Ormond Street Hospital for Children, London, UK; 5 Department of Neurology, Salford Royal NHS Foundation Trust, Manchester, UK; 6 Medical Research Council Center for Neuromuscular Diseases, University College London and National Hospital for Neurology and Neurosurgery, London, UK; 7 Neurogenetics Laboratory, The National Hospital for Neurology and Neurosurgery, London, UK

**Keywords:** neurogenetics, cerebellar ataxia, cerebellar degeneration, neuropathy, genetics

## Letter

Mutations in genes involved in single-strand break repair (SSBR) have been linked to hereditary cerebellar ataxias. Notably, Ataxia-oculomotor apraxia 1 (AOA1 (MIM: 2 08 920)), Spinocerebellar ataxia with axonal neuropathy 1 (SCAN1 (MIM: 6 07 250)) and Ataxia-oculomotor apraxia 4 (AOA4 (MIM: 616267616267616267)) are associated with mutations in *APTX* (MIM: 606350), *TDP1* (MIM: 607198) and *PNKP* (MIM: 605610), respectively.^1^ Recently, compound heterozygous mutations in *XRCC1* were identified in one individual with ocular motor apraxia, cerebellar ataxia and axonal neuropathy.^2^ However, as this study only identified a single case, the causal role of XRCC1 in Autosomal Recessive Cerebellar Ataxia (ARCA) remained in question and required replication in additional cases. Here we recapitulate and broaden this phenotype in two patients of Pakistani decent who were not known to be related. This confirms the role of XRCC1 in cerebellar ataxia.

## Clinical features

**Figure 1:**
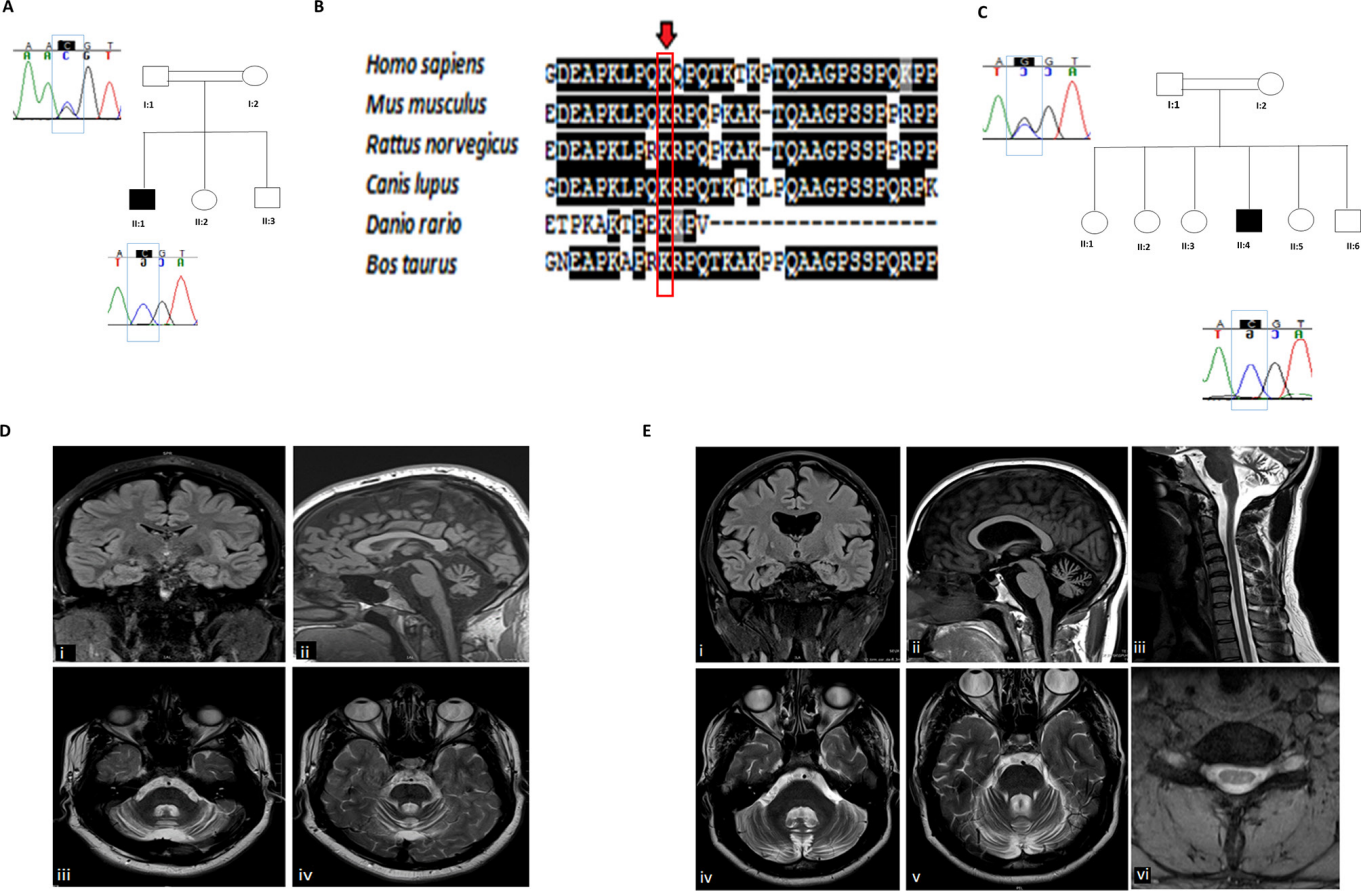
(A) Pedigree of the family of patient 1. Open symbols represent unaffected individuals and filled symbols represent the affected individual. The proband is indicated by an arrow. Sanger sequencing chromatograms surround the symbols and show segregation of mutations. Blue boxes encompass the sites of interest. (B) Structural conservation of the mutated amino acid residue in XRCC1 across six species. Conservation among species of the amino acid residues was determined using Clustal W2 Software for multiple sequence alignment and plotted with BOXSHADE. (C) Pedigree of the family of patient 2. Open symbols represent unaffected individuals and filled symbols represent the affected individual. The proband is indicated by an arrow. Sanger sequencing chromatograms surround the symbols and show segregation of mutations. Blue boxes encompass the sites of interest. (D) Patient 1 : MRI examination of the brain demonstrating preservation of the supratentorial and upper cervical spinal cord neuroparenchymal volumes but disproportionate volume loss of the cerebellum and pons on the on the coronal FLAIR (i) and sagittal T1-weighted (ii) sequences. The axial T2-weighted sequences demonstrate associated volume loss of the middle (iii) and superior (iv) cerebellar peduncles (E) MRI examination of the brain and upper spinal cord. The coronal FLAIR (i) and sagittal T1-weighted (ii) sequences demonstrate mild cortical and subcortical supratentorial and upper spinal cord neuroparenchymal volume loss but disproportionate volume loss of the cerebellum. There is also subtle mild asymmetrical volume loss of the head of the right hippocampus. The axial T2-weighted sequences demonstrate associated volume loss of the middle (iii) and superior (iv) cerebellar peduncles. Sagittal T2-weighted (v) and axial Multiple Echo Recombined Gradient Echo (MERGE) (vi) sequences demonstrate diminution of cervical spinal cord volume for age, particularly evident in the axial anterior–posterior dimension. FLAIR, fluid-attenuated inversion recovery.

*Patient* 1 was the eldest of three healthy siblings from a consanguineous relationship (first cousins) (figure 1A). He walked independently at 15 months. Aged 3 years, he had a persistent ataxic gait and recurrent falls. He experienced impaired fine motor skills and upper limb ataxia. In school, he had mild learning disabilities, dysarthria and difficulties playing sports with painful cramps particularly after prolonged exertion. His examination was notable for prominent bilateral calf wasting and pes planus with impaired coordination and dysdiadochokinesis. Aged 15 years, he underwent an MRI brain, which was unremarkable. Nerve conduction studies and electromyogram (EMG) showed a length-dependent moderately severe sensorimotor neuropathy of axonal type (online supplementary table 1).

10.1136/jnnp-2017-317581.supp1 Supplementary data

Currently, aged 22, he continues to ambulate independently however is prone to frequent falls. Eye examination shows slow saccadic initiation, hypometria, jerky pursuit and horizontal nystagmus on extreme gaze. He has a broad based gait, dysmetria and dysdiadochokinesis. Reflexes are absent in the lower limbs and planters are extensor on the left and equivocal on the right. Sensation to light touch and pain is normal with impaired vibration sense and proprioception. His recent MRI revealed cerebellar atrophy (figure 1D).

*Patient* 2 was also born to consanguineous parents (figure 1C). He had recurrent falls and excessive clumsiness as an infant. Throughout his childhood, he experienced muscle cramps and stiffness in his lower limbs. He was dysarthric and had mild learning difficulties. Diffuse myotonia, predominantly in his hands, led to a diagnosis of myotonia congenita which was confirmed by the identification of a homozygous deletion of exons 17–22 of the *CLCN1* (MIM: 118425) gene.

Now aged 30, he walks without assistance however requires rest after short distances. He has an ataxic waddling gait with diffuse muscle hypertrophy, particularly in the calves and bilateral pes cavus. He has moderate hand and eyelid myotonia. Examination of his eye movements reveal jerky pursuit with nystagmus on lateral gaze. He is areflexic throughout with flexor plantar responses and impaired sensation in the lower limbs below the knee. In addition to his neurological symptoms, he is infertile due to hypogonadism and azoospermia. EMG and MRI revealed a sensorimotor axonal neuropathy (online supplementary table 1) and cerebellar atrophy (figure 1E).

## Whole exome sequencing

The patients and their unaffected parents were recruited with informed consent. DNA was extracted from peripheral blood and whole exome sequencing was carried out on the affected individuals and their parents. Homozygosity mapping was performed identifying three regions of homozygosity shared between the affected individuals including chromosome 1 (236 645 670–237024414), chromosome 8 (90 995 019–95 182 986) and chromosome 19 (42 595 163–45 989 473). Variants within these regions were filtered for rare, homozygous, mutations that were described as deleterious by in silico protein prediction programmes. This identified a homozygous mutation, c.G1293C (p.K431N), in exon 11 of *XRCC1* (NM_006297) in the affected individuals. This mutation lies within a shared haplotype on chromosome 19 and was predicted as deleterious by Sorting Intolerant From Tolerant (SIFT) and possibly damaging by polyphen. The missense mutation c.G1293C (p.K431N) forms part of the donor site of intron 11. Whole exome sequencing was used to establish intrafamilial segregation and confirmed with Sanger sequencing.

## Comment

XRCC1 is a scaffold protein that enables SSBR through interactions with a number of single-strand repair enzymes. Structurally, it comprises three globular domains, an N-terminal and a C-terminal BRCT domain which interact with DNA polymerase and DNA ligase 3α, respectively.^3^ The variant c.G1293C (p.K431N) is within the highly conserved central domain (BRCTa) which facilitates the binding of XRCC1 to ribosylated poly-ADP-ribose polymerase 1 (PARP1) activating partner proteins at sites of single-strand breaks. A loss of functional *XRCC1* results in hyperactivation of PARP.^2^ This likely impedes the access of DNA repair proteins to the break, leading to defective repair and loss of cerebellar neurons. A mouse model with a conditional *XRCC1* deletion illustrated the critical physiological role of *XRCC1* in the development of cerebellar interneurons.^4^

The variant, c.G1293C, exists in the heterozygote state in four individuals of South Asian decent with an allele frequency of 0.0002449 in ExAC. Our patients presented with a slowly progressive cerebellar ataxia and sensorimotor axonal neuropathy, in concordance with the previously described case.^2^ Notably, our patients had a younger age of onset with observable signs at 3 years. Also, their examination elicited comparably subtle ocular signs with slightly prolonged latency in saccadic initiation in only one case.

*XRCC1* is highly expressed in brain particularly in the cerebellum on the GTEX portal, however it is most highly expressed in the pituitary and gonads. This is interesting considering the finding of infertility in patient 2, as in vitro studies of mouse and human sertoli cells demonstrate the involvement of *XRCC1* and *PARP1* (MIM: 173870) in DNA repair which is essential to the survival of these quiescent cells.^5^

In conclusion, homozygous mutations in *XRCC1* result in a core phenotype of slowly progressive cerebellar ataxia accompanied by sensorimotor neuropathy. Expression analysis together with our findings suggest that male infertility may also be a feature of this condition. However, alternative causes have not been ruled out. Therefore, further genotype–phenotype correlation in a larger cohort is warranted. Finally, this variant likely exists at a higher frequency than previously described, particularly in the South Asian population, and should be considered in patients with cerebellar atrophy and peripheral neuropathy from these regions.

## References

[1] Caldecott KW Single-strand break repair and genetic disease. Nat Rev Genet 2008;9:619–31. 10.1038/nrg2380 18626472

[2] Hoch NC, Hanzlikova H, Rulten SL, et al XRCC1 mutation is associated with PARP1 hyperactivation and cerebellar ataxia. Nature 2017;541 10.1038/nature20790 PMC5218588 28002403

[3] Cuneo MJ, London RE Oxidation state of the XRCC1 N-terminal domain regulates DNA polymerase beta binding affinity. Proc Natl Acad Sci U S A 2010;107:6805–10. 10.1073/pnas.0914077107 20351257 PMC2872404

[4] Lee Y, Katyal S, Li Y, et al The genesis of cerebellar interneurons and the prevention of neural DNA damage require XRCC1. Nat Neurosci 2009;12:973–80. 10.1038/nn.2375 19633665 PMC2831284

[5] Ahmed EA, Rijbroek ADB-van, Kal HB, et al Proliferative activity in vitro and DNA repair indicate that adult mouse and human sertoli cells are not terminally differentiated, quiescent cells1. Biol Reprod 2009;80:1084–91. 10.1095/biolreprod.108.071662 19164176

