# Supplementary material for: Mutations in XRCC1 cause cerebellar ataxia and peripheral neuropathy
Source: J Neurol Neurosurg Psychiatry. 2018 Feb 22;89(11):1230–2. doi: 10.1136/jnnp-2017-317581 (PMC6227798; doi:10.1136/jnnp-2017-317581)
Supplement: Supplementary data [file jnnp-2017-317581supp001.pdf]

Table 1: Nerve Conduction Studies of Patient 1 and Patient

Sensory Nerve Conduction Studies			
**Case**	**Nerve**	**Amp (uV)**	**CV (m/s)**
**Patient 1**	**R. Median** (Palm – Wrist)	**8.8**	**57.6**
	**R. Sural** (Calf-Ankle)	Absent	Absent
**Patient 2**	**R. Median** (Palm – Wrist)	**8**	**50**
	**R. Sural** (Calf-Ankle)	**1**	**26**
**Motor Nerve Conduction Studies**			
**Case**	**Nerve**	**CMAP (mV)**	**CV (m/s)**
**Patient 1**	**R. Common Peroneal** (Ankle - EDB)	**3.9**	**35**
	**R. Common Peroneal** (Knee - Ankle)	**6.5**	**40.5**
	**R. Ulnar** (Ab. Elbow – Wrist)	**6.7**	**59.5**
**Patient 2**	**R. Common Peroneal** (Fib. Neck – Ankle)	**5.3**	**37**
	**R. Posterior Tibial** (Pop.Fossa – Ankle)	**0.5**	**40**
	**R. Median** (Wrist - Elbow)	**12.0**	**54**
	**R. Common Peroneal** (Fib. Neck - Ankle)	**5.3**	**37**
